# Prevalence and Predictive Factors of Sickle Cell Emergencies Readmission in the Clinical Hematology Department of Dakar, Senegal

**DOI:** 10.1155/anem/7143716

**Published:** 2024-11-21

**Authors:** Alioune Badara Diallo, Moussa Seck, Sokhna Aissatou Touré, Mohamed Keita, Elimane Seydi Bousso, Blaise Félix Faye, Saliou Diop

**Affiliations:** ^1^Hematology Department, Cheikh Anta Diop University, Dakar, Senegal; ^2^Ouakam Military Hospital, Hematology Department, Cheikh Anta Diop University, Dakar, Senegal

## Abstract

**Introduction:** Sickle cell emergencies are the most common cause of hospitalization for patients with sickle cell disease (SCD). Hospital readmissions represent a considerable financial burden for healthcare systems and increase patient morbidity and mortality. The aim of this study was to assess the prevalence and predictive factors of sickle cell emergencies readmission.

**Materials and Methods:** We conducted a prospective, cross-sectional, descriptive, and analytical study over a 4-month period, including all adult patients admitted for an emergency related to SCD: vaso-occlusive crisis (VOC), acute chest syndrome (ACS), severe anemia, infections, priapism, and stroke. Readmission was considered when the patient returned to the emergency within a period of <30 days, either due to recurrence, persistence of the same complication, or the occurrence of another acute complication related to SCD.

**Results:** We recorded 151 sickle cell emergencies for 112 patients, representing 0.33 emergencies/month/patient. Fifty-eight cases of readmission were recorded, resulting in a readmission rate of 38.41%. Among these patients, 53 (91.37%) had two admissions, and 5 (8.62%) had three admissions. The median age of the patients was 28.41 years (16–70 years), and the sex ratio was 0.57. The SS sickle cell phenotype was predominant in 97 patients (86.61%). The reasons for readmission were VOC (82.75%), ACS (13.72%), and severe anemia (3.44%). The main factors that predicted readmission were the existence of professional activity, a low fetal hemoglobin (HbF) level, the existence of neutrophilia, lymphocytosis, and/or thrombocytosis (*p* values of 0.0084, 0.043, and 0.020 respectively).

**Conclusion:** The 30-day readmission rate after a sickle cell emergency is high in our study. The main factors that predicted readmission were the existence of professional activity, a relatively low level of fetal Hb, the existence of neutrophilia, lymphocytosis, and/or thrombocytosis.

## 1. Introduction

Sickle cell emergencies are acute clinical situations in which the short-term vital and/or functional prognosis is at risk in the absence of appropriate treatment. These are essentially acute complications of sickle cell disease (SCD): vaso-occlusive crisis (VOC), acute chest syndrome (ACS), severe anemia, infections, priapism, and stroke [1, 2]. These are the most frequent causes of hospitalization for sickle cell patients.

Hospital readmissions represent a considerable financial burden for healthcare systems and increase patient morbidity and mortality. The readmission rates of sickle cell emergencies were reported by Brousseau et al. who estimated it at 33.4%, with VOC dominating the causes at 87% [3]. Other studies have also reported high readmission rates and have identified several nonuniform predictive factors, mainly relatively young age, female sex, phenotype, and severity of SCD [4–6]. In sub-Saharan Africa, where the prevalence of SCD is higher, these factors should be identified to develop strategies to reduce sickle cell emergencies readmissions. In this context, we conducted this study, whose aim was to assess the prevalence and predictive factors of sickle cell emergencies readmission in the clinical hematology department of Dakar.

## 2. Materials and Methods

This was a prospective, cross-sectional, descriptive, and analytical study conducted in the hematologic emergency department of the National Blood Transfusion Center in Dakar over a period of 4 months. We included all adult patients admitted for sickle cell emergencies, including VOCs, ACS, severe anemia, infections, priapism, and stroke.

The sociodemographic variables studied were age, sex, marital status, and occupation. Clinical variables included the sickle cell phenotype and reported clinical signs. Biological variables were obtained from emergency tests performed at admission, with a focus on complete blood count (CBC) and inflammation markers, as these parameters are known to correlate with disease exacerbation and can aid in predicting clinical outcomes in patients with SCD.

Hb levels, including HbS and fetal HB (HbF), were assessed using Hb electrophoresis, which provided detailed profiling of Hb types. This method allowed for an accurate estimation of HbS and HbF levels, which are important indicators of disease severity and predictors of clinical outcomes in SCD patients.

A favorable outcome was defined as a good clinical course with normalization of vital signs and clinical examination. An unfavorable outcome was determined if the clinical picture persisted or worsened after emergency treatment, or if the patient died. Readmission was considered if the patient returned to the emergency department within 30 days due to recurrence or persistence of the same complication, or due to another acute complication related to SCD.

The collected data were entered and analyzed using SPSS software. Quantitative variables were expressed as median with interquartile range, and qualitative variables as percentages. Results were presented in tables and graphs. Comparisons were made using the Mann–Whitney *U* test for frequencies and the chi-square test for quantitative variables.

## 3. Results

### 3.1. General Characteristics of Patients

We recorded 151 sickle cell emergencies for 112 patients, representing a median of 0.33 emergencies per month per patient. Sickle cell emergencies accounted for 26.72% of consultations during this period, with a median of two patients admitted per day. Of the patients, 93 (61.59%) had a single admission, 53 (35.1%) had two admissions, and five (3.31%) had three admissions. The reasons for readmission were VOC (48 cases, 82.75%), ACS (8 cases, 13.72%), and severe anemia (2 cases, 3.44%).

The SS sickle cell phenotype was observed in 97 patients (86.61%) (Table 1). The median age of patients was 28 years (range: 16–70 years). The most represented age group was 20–40 years, comprising 77.68% of cases. Female patients predominated, with 71 cases (63.39%), resulting in a sex ratio of 0.57. Seventy-eight patients (69.64%) were unemployed. The most representative professions were pupils and students (44.64%).

Hb electrophoresis showed a median HbF level of 13% for patients with the SS phenotype and 20% for those with S*β*0 thalassemia.

### 3.2. Characteristics of Sickle Cell Emergencies

VOC occurred in 118 cases (78.14%), with triggering factors dominated by infections (56 cases, 37.5%) and physical activity (55 cases, 36.93%). ACS was diagnosed in 16 cases (10.59%), triggered by pneumopathies (11 cases, 67.85%) and persistent VOCs (five cases, 28.57%). Severe anemia was diagnosed in 15 cases (9.93%) due to infectious origins dominated by malaria (11 cases, 69.23%) and pulmonary infections (four cases, 23.07%). Two cases of ischemic stroke were diagnosed, representing 1.34% of the cases (Table 2).

Hydration and analgesics were the most commonly used treatment methods in emergency department.

The clinical outcome was favorable in 68.87% of the cases. The average length of stay in the emergency department (from admission to discharge or transfer to inpatient care) was 3.47 h, with a range from 1 to 6 h.

### 3.3. Frequency of and Reasons for Readmissions to Emergency Departments

Fifty-eight cases of readmission were recorded, resulting in a readmission rate of 38.41%. Among these patients, 53 (91.37%) had two admissions and five (8.62%) had three admissions. The reasons for readmission were VOC (48 cases, 82.75%), ACS (8 cases, 13.72%), and severe anemia (two cases, 3.44%) (Table 3).

### 3.4. Predictive Factors of Readmission

For sociodemographic aspects, no statistically significant differences were observed in readmission rates according to age, sex, or marital status, with *p* values of 0.31, 0.5, and 0.44, respectively. However, the employment status of patients appeared to play a role, as employed sickle cell patients experienced a higher rate of repeat admissions compared to unemployed patients (*p*=0.02). The positive predictive value (PPV) of employment as a factor for readmission risk was approximately 0.41 (41%), suggesting it could be an indirect factor in predicting repeat admissions.

For sickle cell characteristics such as phenotype, vaccination status, and the number of VOCs per year, no significant differences were noted in relation to readmission rates, with *p* values of 0.69, 0.45, and 0.32, respectively. The negative predictive value (NPV) for these characteristics was approximately 0.78 (78%), indicating that their absence did not strongly reduce the likelihood of readmission.

The likelihood of repeat hospital admissions was higher in patients with specific blood abnormalities on the emergency hemogram, demonstrating their predictive potential. Neutrophilia (absolute neutrophil count > 7 G/L) was associated with an increased likelihood of readmission, with an odds ratio (OR) of 1.22, a 95% confidence interval (CI) of [1.02, 1.56], and a *p* value of 0.0084. The PPV of neutrophilia for readmission risk was approximately 0.63 (63%), indicating that patients with elevated neutrophil counts were somewhat more likely to experience repeat admissions. Lymphocytosis (lymphocyte count > 4 G/L) was associated with a higher risk of readmission, with an OR of 2.4, a 95% CI of [1.36, 4.24], and a *p* value of 0.043. The PPV of lymphocytosis reached 0.74 (74%), which suggests that lymphocytosis is a relatively strong predictor of readmission in sickle cell patients. Thrombocytosis (platelet count > 450 G/L) also indicated a higher probability of readmission, with an OR of 1.56, a 95% CI of [1.22, 4.12], and a *p* value of 0.020. The NPV of thrombocytosis, at approximately 0.80 (80%), suggests that the absence of elevated platelet counts may lower the likelihood of readmission, reinforcing its potential role as a predictive marker.

According to Hb electrophoresis parameters, patients with relatively low fetal Hb levels showed a higher frequency of readmissions, with a *p* value of 0.0013. The NPV of higher fetal Hb (HbF) levels was estimated at 0.87 (87%), underscoring the protective role that elevated fetal Hb may play in mitigating the need for hospital readmission in sickle cell patients (Table 4).

## 4. Discussion

SCD is the most widespread genetic disease in the world, with a predominance in sub-Saharan Africa where the prevalence of the gene varies from 10% to 40% (10%-11% in Senegal) [7]. Acute complications of SCD are the most common cause of mortality and the main reason for seeking emergency care in sickle cell patients [8–10].

Our study, which observed readmissions among SCD patients within 30 days following discharge, highlights a concerningly high recurrence of hospital visits due to acute crises. This elevated readmission rate appears notably higher than those observed in some studies from other regions, such as Quebec, as well as in multistate studies conducted in the United States, respectively, at 26.9% and 33.2% [11, 12]. This variance may reflect regional differences in healthcare resources, patient management practices, or environmental factors unique to sub-Saharan Africa.

Readmission rates were consistently distributed throughout the 30-day period following hospital discharge, and the types of diagnoses leading to readmission in the first week were similar to those observed during the entire 30-day period.

The predominance of VOC as the primary reason for readmission aligns with the existing literature, where pain episodes consistently drive healthcare utilization among SCD patients [2, 13]. Our findings also underscore ACS and severe anemia as significant, albeit less frequent, factors for rehospitalization. These complications are well-documented in other studies as central to the SCD emergency profile, suggesting a consistent pattern across diverse settings. However, our study contributes unique insight by identifying specific biological markers associated with higher readmission risk, such as HbF levels and hematologic anomalies such as neutrophilia and thrombocytosis. These markers may indicate an underlying inflammatory or infectious process, which, if undiagnosed or poorly managed, could contribute to recurrent crises.

Employment status also emerged as a predictor of readmission in our cohort, possibly reflecting the physical strain that can exacerbate SCD symptoms. Physical exertion, particularly in occupational settings, likely induces hypoxia and dehydration, both recognized triggers of VOCs [1, 13]. This observation highlights the need for tailored patient education on managing activity levels to reduce the crisis frequency.

The association of HbF with increased readmission risk supports the widely accepted role of HbF in moderating SCD severity. Higher HbF levels are known to inhibit Hb S polymerization, which may reduce the frequency and intensity of pain episodes [3, 14]. Therefore, therapies aimed at increasing HbF, such as hydroxyurea, could be instrumental in reducing recurrent admissions in our patient population.

In addition, the presence of neutrophilia and thrombocytosis in our readmitted patients points to a potential role of subclinical infections or inflammatory responses in precipitating SCD crises.

Neutrophilia is a marker of infection in many clinical contexts. However, it should be noted that modest neutrophilia is often observed in sickle cell patients, even in a stable state in the absence of infection, often explained by a redistribution of neutrophils from the marginal pool to the circulating pool. However, the prevalence and intensity of neutrophilia is higher in sickle cell patients with bacterial infections compared to those without infection [15].

Thrombocytosis is a common phenomenon in sickle cell patients, even in a stable state and in the absence of any infection. This has been attributed to chronic hemolytic anemia and asplenia [16]. However, previous studies have shown that sickle cell patients with infected patients had more intense thrombocytosis compared to their counterparts without infections. This finding has been interpreted as reflecting the additional effect of reactive inflammatory changes associated with infection [17].

These hematologic markers reflect the inflammatory state during acute complications of SCD and should raise the possibility of infection. Neutrophilic polynucleosis and thrombocytosis, which are predictive factors for readmission, are probably related to underlying infections that are not recognized during emergency clinical examination and therefore are not managed. Infections are the main trigger for sickle cell crises, particularly in sub-Saharan Africa [18, 19].

In summary, the factors identified in our study as predictors for readmission are largely modifiable. Effective strategies could include patient education on activity modification, the use of therapies to increase HbF levels, and enhanced screening for infections during acute crises. These preventive measures could help reduce the frequency of hospital readmissions, ultimately improving quality of life and reducing the healthcare burden for individuals with SCD.

## 5. Conclusion

This study aimed to identify predictive factors for a 30-day readmission following sickle cell emergencies, with the goal of better understanding and mitigating risks associated with recurrent hospitalizations. Key predictors identified include employment status, HbF levels, and specific hematologic markers such as neutrophilia, lymphocytosis, and thrombocytosis. Recognizing these factors enhances our ability to stratify risk and target interventions more effectively. By focusing on modifiable elements such as activity levels, use of hydroxyurea, and infection management, healthcare providers can develop strategies that potentially reduce readmission rates and improve patient outcomes in SCD.

## Figures and Tables

**Table 1:** General characteristics of patients and number of admissions.

Parameters	Headcounts	Frequency (%)
Age groups		
< 20 years	14	12.5
20–40 years	87	77.68
≥ 40 years	11	9.82
Sex		
Men	41	36.6
Women	71	63.39
Employments (sectors of activity)		
Unemployed	78	69.64
Formal sector	12	10.71
Informal sector	22	19.64
Sickle cell phenotype		
SS	97	86.61
S*β*0 thalassemia	8	7.14
SC	4	3.57
S*β* + thalassemia	3	2.68
Number of admissions		
1 admission	93	61.59
2 admissions	53	35.1
3 admissions	5	3.31
Total number of readmissions	58	3.84

**Table 2:** Type and frequency of sickle cell emergencies.

Sickle cell emergencies	Headcounts	Frequency (%)
VOC	118	78.14
ACS	16	10.59
Severe anemia	15	9.93
Stroke	2	1.34

**Table 3:** Reasons for readmissions.

Reasons for readmission	Headcounts	Percentage (%)
VOC	48	82.75
ACS	8	13.72
Severe anemia	2	3.44

**Table 4:** Biological factors associated with readmission.

	1 admission	≥ 2 admissions	*p* value
Blood count parameters (median [IQR])			
Hb (g/dL)	7.35 [6.5, 9.8]	6.8 [6.1, 8.7]	0.31
MCV (fL)	85.00 [79.60, 89.80]	80.60 [76.25, 86.88]	0.17
MCHC (g/dL)	35.20 [34.25, 35.95]	34.80 [33.88, 35.62]	0.51
Leucocytes (G/L)	13.27 [10.05, 15.89]	12.66 [9.39, 16.96]	0.084
Lymphocytes (G/L)	3.20 [2.62, 4.00]	2.38 [2.04, 3.00]	0.043∗
Neutrophiles (G/L)	7.94 [5.47, 11.37]	10.25 [7.59, 13.13]	0.0084∗
Monocytes (G/L)	1.32 [0.82, 1.64]	0.89 [0.81, 1.31]	0.41
Platelets (G/L)	367.50 [289.75, 472.25]	445.50 [408.25, 540.25]	0.020∗
Hemoglobin electrophoresis			
Hb S	84.45 [78.67, 89.53]	79.20 [68.10, 85.80]	0.2
Hb A2	2.80 [2.32, 3.30]	2.40 [1.80, 3.10]	0.393
Hb F	11.20 [4.90, 15.50]	18.70 [13.30, 29.90]	0.0013∗
Total (*n*)	93	58	

^∗^Refer to parameters with a significative *p* value.

## Data Availability

The data that support the findings of this study are available from the corresponding author upon reasonable request.
